# Differential Targeting of Gr-MDSCs, T Cells and Prostate Cancer Cells by Dactolisib and Dasatinib

**DOI:** 10.3390/ijms21072337

**Published:** 2020-03-27

**Authors:** Guoqiang Liu, Zhijian Jin, Xin Lu

**Affiliations:** 1 Integrated Biomedical Sciences Graduate Program, University of Notre Dame, Notre Dame, IN 46556, USA; gliu4@nd.edu; 2 Department of Biological Sciences, Boler–Parseghian Center for Rare and Neglected Diseases, University of Notre Dame, Notre Dame, IN 46556, USA; zjin2@nd.edu; 3 Harper Cancer Research Institute, University of Notre Dame, Notre Dame, IN 46556, USA; 4 Tumor Microenvironment and Metastasis Program, Indiana University Melvin and Bren Simon Cancer Center, Indianapolis, IN 46202, USA

**Keywords:** granulocytic myeloid-derived suppressor cells, immune checkpoint blockade, combination immunotherapy, castration-resistant prostate cancer, Dactolisib, Dasatinib, reverse phase protein array, PI3K/mTOR, Src, mitochondrial respiration

## Abstract

Granulocytic myeloid-derived suppressor cells (Gr-MDSCs) promote immune evasion and resistance to immunotherapeutics in a variety of malignancies. Our previous study showed that dual PI3K/mTOR inhibitor Dactolisib impaired the viability and immunosuppressive function of Gr-MDSCs, and significantly synergized with immune checkpoint blockade (ICB) antibodies targeting PD1 and CTLA4 to eradicate metastatic castration-resistant prostate cancer (CRPC) in a preclinical transgenic mouse model. On the contrary, tyrosine kinase inhibitor Dasatinib diminished tumor-infiltrating T lymphocytes and showed no synergic activity with ICB. The understanding of the distinct effects of Dactolisib and Dasatinib on Gr-MDSCs, T cells and prostate neoplastic cells is inadequate, limiting the clinical translation of the combination immunotherapy. To address this question, we applied Reverse Phase Protein Array (RPPA) to profile 297 proteins and protein phosphorylation sites of Gr-MDSCs, T cells and prostate cancer cells isolated from the CRPC model. We found cell type-specific protein expression patterns and highly selective targets by the two drugs, including preferential inhibition of phospho-4E-BP1 in Gr-MDSCs by Dactolisib and preferential suppression of phospho-Src and phospho-p38 MAPK in T cells. Furthermore, transcriptomic profiling of Gr-MDSCs treated with the two inhibitors revealed downregulation of mitochondrial respiration pathways by Dactolisib but not Dasatinib. Overall, these results provide important mechanistic insight into the efficacious combination of Dactolisib and ICB as well as the detrimental effect of Dasatinib on anti-tumor immunity.

## 1. Introduction

The microenvironment of solid tumors is comprised of a complex mixture of epithelial cells, stromal cells, endothelial cells and immune cells [1]. Among tumor-infiltrating immune cells, myeloid-derived suppressor cells (MDSCs) represent a cell type gaining tremendous interest in recent years. MDSCs are a heterogeneous population of immature myeloid cells with differentiation programs partially blocked in pathological conditions such as cancer [2]. MDSCs can be phenotypically divided into granulocytic (Gr-MDSCs, CD11b^+^ Ly6G^+^ Ly6C^low^ in mouse, CD11b^+^ CD14^−^ CD15^+^ CD66b^+^ in human) and monocytic (Mo-MDSCs, CD11b^+^ Ly6G^−^ Ly6C^high^ in mouse, CD11b^+^ CD14^+^ CD15^−^ HLA-DR^−/low^ in human) subpopulations [3]. Through inhibition of T cells, especially cytotoxic T lymphocytes (CTLs), MDSCs play important tumor-promoting roles and maintain a state of immunological anergy and tolerance [4,5]. MDSCs can suppress T cell activity through deprivation of nutrients, such as L-arginine and L-cysteine, and interference with T cell receptor function and downstream signal pathways via reactive oxygen species and reactive nitrogen species [6,7]. 

Immunosuppression by MDSCs may have profound implications in cancer immunotherapy. Among the immunotherapeutic modalities, immune checkpoint blockade (ICB) to reinvigorate CTLs using antibodies targeting CTLA4 or PD1/PD-L1 generates durable therapeutic responses in patients across a variety of cancer types [8,9]. However, some cancers such as metastatic castration-resistant prostate cancer (CRPC) show de novo resistance to ICB [10,11]. Prostate cancer (PCa) microenvironment is highly immunosuppressive and infiltrated with abundant MDSCs, especially Gr-MDSCs as the predominant population in both mouse models and clinical PCa [12,13,14,15], suggesting Gr-MDSCs as the major barrier to block the efficacy of ICB. 

Previously, we reported that, in the *PB-Cre^+^ Pten^L/L^ p53^L/L^ Smad4^L/L^ mTmG^L/+^ LSL-Luc^L/+^* (CPPSML) transgenic mouse model of metastatic CRPC, ICB therapy could be effectively improved through pharmacological targeting of Gr-MDSCs [16]. Specifically, while CRPC developed in the CPPSML model responded poorly to either the ICB antibody cocktail composed of anti-PD1 and anti-CTLA4 or the PI3K/mTOR dual inhibitor Dactolisib (as known as BEZ235), the combination of these agents elicited a strong synergistic effect on eradicating both the primary and metastatic CRPC [16]. Mechanistically, Dactolisib inhibited the viability and immunosuppressive activity of Gr-MDSCs through silencing the PI3K signaling and upregulation of interleukin-1 receptor antagonist while sparing the activity of CD4^+^ and CD8^+^ T cells, thus creating a tumor microenvironment permissive to the effect from ICB on unleashing CTLs. On the contrary, the tyrosine kinase inhibitor (TKi) Dasatinib was incapable of cooperating with ICB because of its potent activity to diminish tumor-infiltrating T cells [16], consistent with the reported Dasatinib inhibition of T cell receptor-mediated signal transduction and proliferation [17]. 

Despite this previous study, we have inadequate understanding of the differential effect of Dactolisib and Dasatinib on Gr-MDSCs, T cells and PCa cells at the protein levels. To address this, we isolated these cell types from the CPPSML model, applied a short in vitro treatment (2 h) with Dactolisib or Dasatinib, and subjected the cells to the targeted proteomic profiling with Reverse Phase Protein Array (RPPA). RPPA technology is a high-throughput dot-blot immunoassay to provide semi-quantitative measurement of total protein levels and post-translational modifications (PTMs) across a variety of signaling pathways involved in cancer and immunology [18]. In our study, the RPPA platform included 297 unique antibodies, which demonstrated distinct protein expression patterns for Gr-MDSCs, T cells and PCa cells. We found that each cell type displayed specific responses to Dactolisib and Dasatinib at the protein level, validated further by western blot. Furthermore, to examine the effect of the two drugs on the transcriptome of Gr-MDSCs, the 6 h treated cells were profiled by microarray, which revealed downregulation of mitochondria-related pathways by Dactolisib but not Dasatinib treatment. These results together provide critical insights into the disparate effects of these two drugs when used together with ICB in metastatic CRPC. 

## 2. Results

### 2.1. Distinct Protein Expression Pattern by PCa Cells, T Cells and Gr-MDSCs in a Mouse CRPC Model 

**Figure 1:**
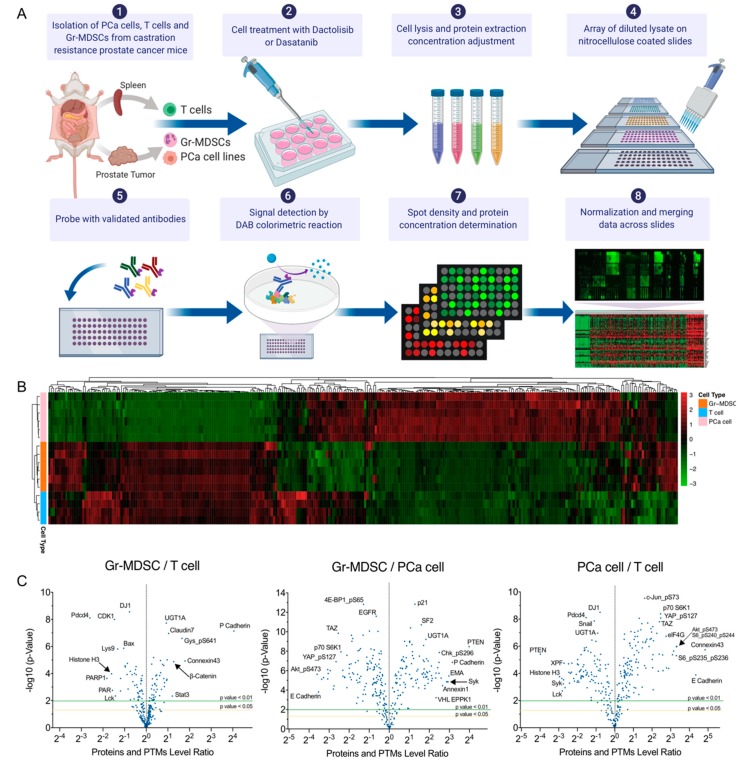
RPPA profiling of PCa cells, Gr-MDSCs and T cells of the CPPSML model of CRPC. (**A**) Workflow of cell isolation and RPPA. The figure was created with BioRender.com. (**B**) Unsupervised hierarchical clustering of PCa cells, T cells and Gr-MDSCs (untreated or DMSO-treated) with normalized RPPA signals. Each row represents a biological replicate sample and each column corresponds to an antibody in the RPPA array. (**C**) Volcano plots showing the pairwise fold change comparisons of the normalized protein or PTM levels among the three cell types. *p* values were calculated by Student’s *t*-test. Arrows help to pinpoint proteins of interest of whose labels were partly overlapped with adjacent labels.

In the same procedure as we reported [16], we induced CRPC formation in CPPSML model by surgically castrating CPPSML males when prostate tumors reached 150 mm^3^ measured by magnetic resonance imaging, followed by feeding the mice with an enzalutamide-admixed diet for 4 weeks. At this stage, the mice were euthanized and the prostate tumors were dissected and digested for isolation of primary PCa cells using fluorescence-activated cell sorting (FACS) of GFP^+^ CD45^−^ cells, or isolation of tumor-infiltrating Gr-MDSCs using magnetic-activated cell sorting (MACS) of CD11b^+^ Ly6G^+^ Ly6C^low^ cells. From the same mice, total T cells were isolated from the spleen using MACS. PCa cells were cultured for 2–3 passages as adherent primary cells before inhibitor treatment, whereas Gr-MDSCs and T cells were treated immediately after isolation to maximize survival. Cells were treated with DMSO (control), Dactolisib or Dasatinib at various concentrations for 2 h before harvest for the RPPA workflow (Figure 1A, Supplementary Table S1). Unsupervised clustering of the log2 transformed RPPA signals of untreated or DMSO-treated cell samples (6 PCa cell samples, 6 Gr-MDSC samples, 4 T cell samples) grouped the cells in accurate concordance with their cell types (Figure 1B), indicating the distinct expression pattern of the three cell types. It is readily noticeable that T cells and Gr-MDSCs share more similarity in comparison with PCa cells, consistent with the fact that the former two cell types are both descendants of hematopoietic lineage, whereas PCa cells are of epithelial lineage. Differential expression analysis of PCa cells, T cells and Gr-MDSCs, shown in volcano plots, demonstrate interesting patterns (Figure 1C, Supplementary Table S2–S4). For example, Gr-MDSCs have a unique high level of P-cadherin over both T cells and PCa cells (Gr-MDSC/T = 16.6, Gr-MDSC/PCa = 8.89). T cells highly express Histone-H3 and Lck compared with Gr-MDSCs (T/Gr-MDSC for Histone-H3 = 3.04, T/Gr-MDSC for Lck = 2.68) and PCa cells (T/PCa for Histone-H3 = 7.76, T/PCa for Lck = 7.01). PCa cells have a higher abundance of a number of proteins and PTMs compared with Gr-MDSCs and T cells, such as E-cadherin, Connexin-43, ARID1A, eIF4G, HES1, phospho-Akt (Ser473), phospho-S6 (Ser235/236, Ser240/244), Phospho-NDRG1 (Thr346), phospho-YAP (Ser127), and TAZ (fold change > 5). Enrichment of phospho-Akt and phospho-S6 in PCa cells is consistent with the prostate-specific deletion of *Pten* in the CPPSML model. 

### 2.2. Differential Effect of Dactolisib and Dasatinib on Protein Phosphorylation in PCa Cells, T cells and Gr-MDSCs

**Figure 2:**
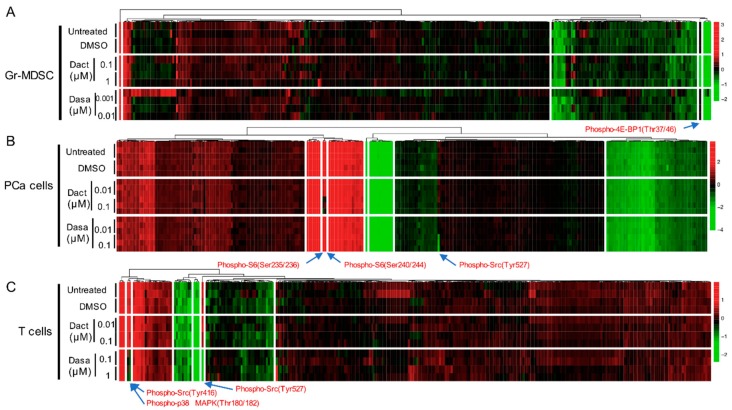
Heatmaps showing the normalized protein expression signals under no treatment or 2 h treatment of DMSO, Dactolisib or Dasatinib (at indicated concentration) for Gr-MDSCs (**A**)**,** PCa cells (**B**), and T cells (**C**). Proteins with significant changes under either inhibitor treatment are labeled with arrows. Dact = Dactolisib, Dasa = Dasatinib. Arrows highlight the differentially expressed proteins or PTMs by the inhibitor treatment.

To identify the treatment effect of Dactolisib and Dasatinib on specific proteins or PTMs in the three cell types, we generated supervised clustering trees of the normalized RPPA data of the control (untreated and DMSO-treated) and inhibitor-treated (Dactolisib or Dasatinib) samples. Dysregulated targets emerged in cell type-specific and inhibitor-specific manners. For Gr-MDSCs, among the 297 unique antibodies, only phospho-4E-BP1 (Thr37/46) was consistently decreased by Dactolisib, but it was unaffected by Dasatinib (Figure 2A), consistent with the biological effect of Dactolisib on inhibiting PI3K/mTOR signaling. In a similar manner, Ser235/236 and Ser240/244 phosphorylation of S6 in PCa cells were downregulated by Dactolisib but remained unchanged under Dasatinib treatment (Figure 2B). By contrast, phospho-Src (Tyr527) was only downregulated by Dasatinib in PCa cells (Figure 2B), corresponding to the activity of Dasatinib as a Src inhibitor [19]. For T cells, Dasatinib, but not Dactolisib, effectively downregulated phospho-Src (Tyr416 and Tyr527) and phospho-p38 MAPK (Thr180/182) (Figure 2C). Together, these results demonstrate that with a short time treatment (2 h), Dactolisib and Dasatinib attenuated specific signaling targets of the PI3K/mTOR or Src/MAPK pathway, respectively. It is interesting to note that the majority of the surveyed proteins and PTMs remained largely unchanged by the inhibitors in all three cell types.

### 2.3. Cell Type-Specific Downregulation of Phosphorylation Levels of S6, 4E-BP1, Src and p38 MAPK by Dactolisib and Dasatinib

**Figure 3:**
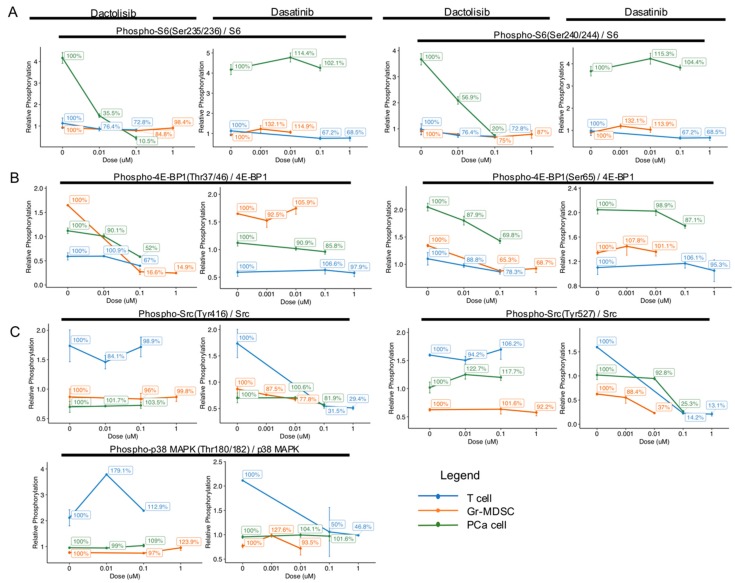
Cell type-specific downregulation of phosphorylation levels of S6, 4E-BP1, Src and p38 MAPK by Dactolisib and Dasatinib. Phosphorylation level of the specified proteins was normalized to total protein level, and relative percentage was calculated with no drug treatment (0 μM) as 100%. (**A**) The phosphorylation of S6 at Ser235/236 and Ser240/244 dramatically decreased in PCa cells by Dactolisib, while Dasatinib had little effect. (**B**) The phosphorylation of 4E-BP1 at Thr37/46 in Gr-MDSCs was dramatically decreased by Dactolisib. The phosphorylation of 4E-BP1 at Ser65 in Gr-MDSCs, T cells and PCa cells was mildly decreased by Dactolisib. Dasatinib had little effect. (**C**) The phosphorylation of Src at Tyr416 and Tyr527 and p38 MAPK at Thr180/182 was dramatically decreased in T cells by Dasatinib, but not Dactolisib. In each plot, data represent mean ± standard error of the mean.

In order to compare the dose-dependent effect of Dactolisib and Dasatinib on different cell types, we calculated the ratio of phosphorylated over total protein RPPA signal intensity for S6, 4E-BP1, Src and p38 MAPK (identified above as differentially regulated by the inhibitors) and plotted for each cell type at the tested concentrations (Figure 3). For each cell type, the ratio at different inhibitor concentrations was normalized to DMSO control (0 μM) for each cell type and expressed as percentages to help show the relative changes (Figure 3). 

For the ratio of phospho-S6 / total S6, Dactolisib dramatically decreased phosphorylation of Ser235/236 (100% at 0 μM, 10.5% at 0.1 μM) and Ser240/244 (100% at 0 μM, 20% at 0.1 μM) only in PCa cells, but not in T cells or Gr-MDSCs (Figure 3A). On the other hand, Dasatinib caused little effect on phospho-S6 in either cell type. The higher basal level of phospho-S6 and higher sensitivity to Dactolisib in PCa cells compared with T cells or Gr-MDSCs is consistent with the *Pten*-deficient status of PCa cells. 

For the ratio of phospho-4E-BP1 / total 4E-BP1, Dactolisib mildly repressed phosphorylation at Thr37/46 and Ser65 in all cell types (100% at 0 μM, >50% at 0.1 μM), except for Thr37/46 in Gr-MDSCs (100% at 0 μM, 16.6% at 0.1 μM, Figure 3B). Similar to the effect on phospho-S6, Dasatinib caused marginal effects on phospho-4E-BP1 (Thr37/46 or Ser65) in either type of cells.

For the phosphorylation of Src and p38 MAPK, we noticed that Dactolisib generated little effect on all three cell types (100% at 0 μM, >95% at 0.1 μM, Figure 3C). On the contrary, Dasatinib showed particularly strong inhibition on phosphorylation of Src and p38 MAPK in T cells: phospho-Src Tyr416 reduced from 100% to 31.5% at 0.1 µM, phospho-Src Tyr527 reduced from 100% to 14.2% at 0.1 µM, and phospho-p38 MAPK Thr180/182 reduced from 100% to 50% at 0.1 µM (Figure 3C). Effect of Dasatinib on phospho-Src Tyr416 and phospho-p38 MAPK Thr180/182 in PCa cells and Gr-MDSCs was moderate to minimal, but its effect on phospho-Src Tyr527 in PCa cells and Gr-MDSCs was substantial (reduced from 100% to 25.3% at 0.1 µM in PCa cells, and from 100% to 37% at 0.01 µM in Gr-MDSCs, Figure 3C). 

**Figure 4:**
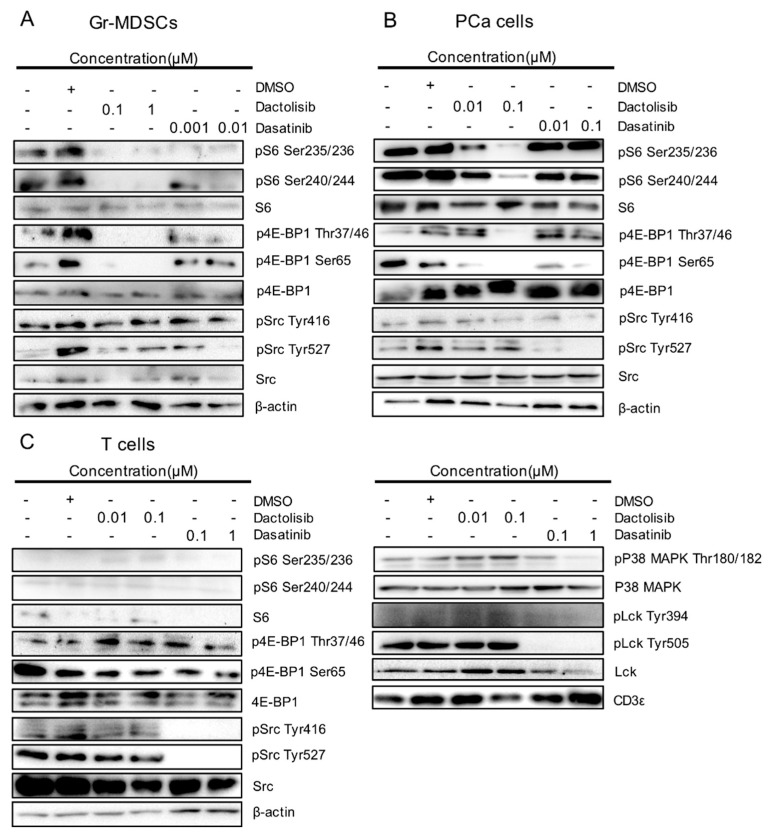
Validation of cell type-specific effect on S6, 4E-BP1, Src and p38 MAPK by Dactolisib and Dasatinib. Cell lysates used for RPPA were used to detect indicated total proteins and their phosphorylated forms by western blot for (**A**) Gr-MDSCs, (**B**) PCa cells and (**C**) T cells. The sign + and – denote the presence and absence of DMSO, Dactolisib or Dasatinib, respectively.

To corroborate the results from RPPA, we detected the dysregulated proteins and PTMs by western blot. Dactolisib strongly inhibited S6 phosphorylation at Ser235/236 and Ser240/244 in Gr-MDSCs and PCa cells (Figure 4A,B). S6 and phospho-S6 were barely detectable in T cells (Figure 4C). Dactolisib also suppressed 4E-BP1 phosphorylation at Thr37/46 and Ser65 in Gr-MDSCs and PCa cells (Figure 4A,B), yet 4E-BP1 phosphorylation in T cells was largely resistant to Dactolisib treatment. In contrast to Dactolisib, the effect of Dasatinib on phosphorylation of S6 or 4E-BP1 in all three cell types was much weaker or essentially absent (Figure 4A–C). However, Dasatinib (but not Dactolisib) inhibited p38 MAPK phosphorylation at Thr180/182, Src phosphorylation at both Tyr416 and Try527 and Lck phosphorylation at both Tyr394 and Tyr505, preferentially in T cells (Figure 4C). As an internal control, CD3ε remained unchanged in T cells by the drug treatments (Figure 4C). It is worth noting that the moderate inhibitory effect of Dasatinib on Src phosphorylation in Gr-MDSCs and PCa cells was only observed for Tyr527 but not Tyr416 (Figure 4A,B). Overall, these results validated the RPPA results and supported (1) the selective downregulation of phospho-S6 and phospho-4E-BP1 by Dactolisib in Gr-MDSCs and PCa cells, and (2) the preferential downregulation of phospho-Src and phospho-p38 MAPK by Dasatinib in T cells.

### 2.4. Dactolisib, but not Dasatinib, Elicited Substantial Transcriptomic Alteration of Gr-MDSCs and Downregulated Genes Associated with Mitochondrial Respiration

**Figure 5:**
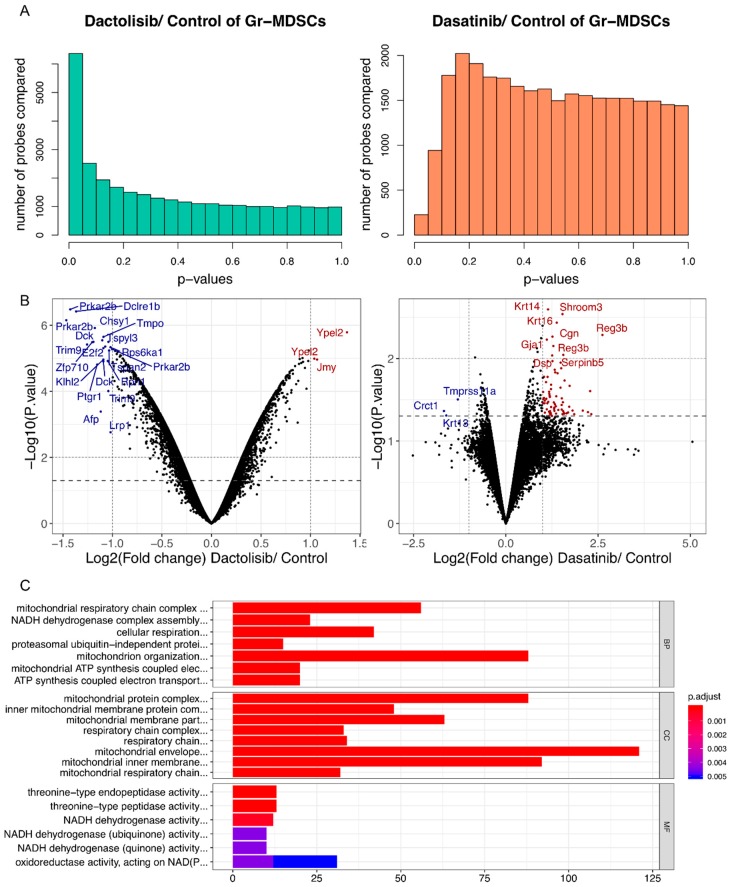
Dactolisib, but not Dasatinib, downregulated mitochondrial respiration genes in Gr-MDSCs based on microarray analysis. (**A**) *p* value distributions for differentially expressed probes between Dactolisib and DMSO control, or between Dasatinib and DMSO control based on unpaired *t*-test. (**B**) Volcano plots showing differentially expressed genes by Dactolisib or Dasatinib treatment on Gr-MDSCs. Some of the most significantly altered genes are labeled. Blue/Red: downregulated/upregulated genes by inhibitor treatment. (**C**) GO term over-representation analysis of downregulated genes by Dactolisib treatment, highlighting the enrichment of genes involved in mitochondrial respiration activity. BP: biological process; CC: cellular component; MF: molecular function.

To provide a complementary view of the gene expression changes by Dactolisib and Dasatinib treatment in Gr-MDSCs, we treated CRPC-derived Gr-MDSCs with DMSO (*n* = 4), Dactolisib (0.1 μM, *n* = 2) or Dasatinib (0.1 μM, *n* = 2) for 6 h before purifying the mRNA for microarray profiling. Differential expression analysis identified more genes with altered expression by Dactolisib treatment (1733 genes upregulated, 2663 genes downregulated, *p* < 0.05; 676 genes upregulated, 1414 downregulated, *p* < 0.01) than Dasatinib treatment (154 genes upregulated, 46 genes downregulated, *p* < 0.05; 11 genes upregulated, 12 downregulated, *p* < 0.01) (Figure 5A,B, Figure S1A, Supplementary Tables S5 and S6). To explore the functional consequence of the altered gene expression, each of the 4 lists of altered genes (Dactolisib-upregulated, *p* < 0.01; Dactolisib-downregulated, *p* < 0.01; Dasatinib-upregulated, *p* < 0.05; Dasatinib-downregulated, *p* < 0.05) were analyzed for Gene Ontology (GO) over-representation, KEGG pathway enrichment and Reactome enrichment. Only the Dactolisib-downregulated genes showed significantly enriched GO terms and pathways. The enriched GO terms were mainly associated with mitochondrial functions, such as mitochondrial respiration chain complex assembly, ATP synthesis coupled electron transport, and NADH dehydrogenase complex assembly (Figure 5C, Supplementary Table S7). Similarly, oxidative phosphorylation was the top-ranked enriched pathway in KEGG analysis (Figure S1B, Supplementary Table S8), and respiratory electron transport/ATP synthesis by chemiosmotic coupling was the top-ranked enriched pathway by Reactome analysis (Figure S1C, Supplementary Table S9). No significant enrichment was found for Dactolisib-upregulated, Dasatinib-upregulated, and Dasatinib-downregulated genes.

## 3. Discussion

Our study interrogates the differential effect of Dactolisib and Dasatinib on PCa cells, T cells and Gr-MDSCs isolated from the same mouse primary CRPC. Employing the power of RPPA, this study reveals the unique protein expression patterns of the three cell types. Some of the features are expected, such as higher Lck in T cells and higher E-cadherin, phospho-Akt and phospho-S6 in PCa cells. The dramatically higher P-Cadherin level in Gr-MDSCs compared with T cells and PCa cells is surprising and intriguing. As a calcium-dependent cell–cell adhesion glycoprotein, P-Cadherin shares about 67% of homology with E-Cadherin, yet remains far less characterized [20]. P-Cadherin is expressed by myoepithelial/basal cells in a heterogeneous manner in organs like the basal layer of the epidermis, breast, prostate, and part of the digestive tract [20]. Normal hematopoietic cells are not known to express P-Cadherin. Given that Gr-MDSCs massively dysregulate gene expression relative to normal neutrophils [21,22], it will be important to investigate whether human Gr-MDSCs also upregulate P-Cadherin and the role of P-Cadherin in the biological function of Gr-MSDCs. Once validated, P-Cadherin may be a new surface marker and therapeutic target for Gr-MDSCs. P-Cadherin^+^ cells can be targeted for killing using P-Cadherin-targeted radioimmunotherapeutic FF-21101(^90^Y) [23] or antibody-drug conjugates [24]. 

Upon the 2 h drug treatment, PCa cells, T cells and Gr-MDSCs all exhibit rather limited changes of protein levels and protein phosphorylation levels. This result can be accounted for by two reasons: first, among the proteins and PTMs identifiable by the 297 unique antibodies, Dactolisib and Dasatinib were highly selective and only affected a few targets; second, the short drug incubation duration restricted changes only to the direct targets and more broad secondary effects were not detected. The changes caused by the two inhibitors on PCa cells, T cells and Gr-MDSCs highlight the specificity of Dactolisib and Dasatinib: the dual PI3K/mTOR inhibitor Dactolisib dampened phospho-S6 and phospho-4E-BP1, whereas the TKi Dasatinib attenuated phospho-Src and phospho-p38 MAPK. 

The main significance of this study is that through the distinct regulation of the two drugs on PCa cells, T cells and Gr-MDSCs, we can gain insights into the mechanism of action of Dactolisib and Dasatinib and help explain why Dactolisib can, but Dasatinib cannot, cooperate with ICB therapy in treating preclinical CRPC [16]. First, Dactolisib preferentially inhibited phospho-S6 (Ser235/236, Ser240/244) in PCa cells and phospho-4E-BP1 (Thr37/46) in Gr-MDSCs (Figure 3A,B). Dactolisib inhibits PI3K and mTOR kinase activity by binding to the ATP-binding cleft of these enzymes [25]. mTOR is the key component of mTORC1 complex that promotes protein synthesis largely through the phosphorylation of two effectors, p70 S6 kinase (S6K1, which further phosphorylates S6) and 4E-BP1 [26]. Previous studies demonstrate that S6K1 and 4E-BP1 can be dephosphorylated in different patterns by the same mTORC1 inhibitor (e.g., rapamycin) in a cell type-dependent manner [27,28]. In the CPPSML PCa cells, *Pten* deficiency leads to hyperactive PI3K/mTOR signaling [16,29], whereas for Gr-MDSCs, several studies have established the essential role of PI3Kδ/γ signaling in the immunosuppressive function [16,30,31]. Due to the differences in upstream activation mechanisms, it is conceivable that mTORC1 in the two cell types elicits differential activation of S6K1 and 4E-BP1, which may explain the differential response of phospho-S6 and phospho-4E-BP1 in the two cell types to the same pan-PI3K inhibitor Dactolisib. The much more significant reduction of Thr37/46 phosphorylation of 4E-BP1 compared with Ser65 phosphorylation in Dactolisib-treated Gr-MDSCs is likely caused by the hierarchical phosphorylation process of 4E-BP1, whereby phosphorylation of Thr37/46 is a priming event for the subsequent phosphorylation of Ser65 [32,33]. It will be interesting for future experiments to define the role of 4E-BP1 phosphorylation in the immunosuppressive function of Gr-MDSCs. 

With these said, our validation studies with western blot still revealed substantial downregulation of both phospho-S6 and phospho-4E-BP1 by Dactolisib treatment in Gr-MDSCs (Figure 4A), consistent with our previous publication [16]. Moreover, at the transcriptomic level, Dactolisib treatment (but not Dasatinib) on Gr-MDSCs caused an interesting downregulation of genes involved in the mitochondrial electron transport chain (Figure 5C, Figure S1B,C). It is known that mTOR signaling coordinates energy consumption by regulating mRNA translation and synthesis of nucleus-encoded mitochondria-related components of complexes I and V [34]. Specifically, mTORC1 controls mitochondrial activity and biogenesis by selectively promoting translation of nucleus-encoded mitochondria-related mRNAs via inhibition of 4E-BP [35]. A previous study demonstrated reprogrammed metabolism in tumor-infiltrating MDSCs in various murine tumor models, whereby these cells upregulated fatty acid uptake and β oxidation, mitochondrial biogenesis and oxygen consumption [36]. Inhibition of fatty acid oxidation by etomoxir attenuated the immunosuppressive activity of MDSCs and synergized with immunotherapy [36], similar to our observation of the synergistic anti-tumor efficacy of Dactolisib and immunotherapy [16]. Collectively, these results suggest that a major effect of Dactolisib on Gr-MDSCs is inhibition of mTOR and oxidative phosphorylation, which compromises fatty acid oxidation and leads to loss of immunosuppression.

Dasatinib preferentially inhibited Src and p38 MAPK phosphorylation in T cells, providing a molecular explanation for the effect of Dasatinib on diminishing T cells in the CRPC microenvironment [16]. Src-family kinases Lck and Fyn in proximal T-cell receptor signal transduction play critical roles in T-cell activation, differentiation, and tolerance [37]. Dasatinib inhibits Src, Lck and Fyn [38]. While the RPPA platform did not have antibodies recognizing phospho-Lck, Fyn or phospho-Fyn, the antibodies for Src and phospho-Src (Tyr527, Tyr416) provided surrogates for evaluating the effects of Dasatinib on T cells as well as PCa cells and Gr-MDSCs. Src activity is regulated by tyrosine phosphorylation at two sites, but with opposing effects [39]. Phosphorylation at Tyr527 in the carboxy-terminal tail by Csk renders Src in a closed, inactive conformation. Upon dephosphorylation of Tyr527, Src changes into an open conformation and self-phosphorylates Tyr416 to become activated [39]. Dasatinib reduced Tyr527 phosphorylation in all three cell types, yet only reduced Tyr416 phosphorylation in T cells (Figure 3C, Figure 4C), suggesting complete loss of Src activity preferentially in T cells. Additional validation experiments with western blot showed dampened Lck phosphorylation by Dasatinib in T cells (Figure 4C), which further helps explain the depletion of tumor-infiltrating T cells by a month-long Dasatinib treatment in the CPPSML CRPC model [16]. Moreover, Dasatinib preferentially inhibited the phosphorylating activation of p38 MAPK (Figure 3C, Figure 4C), a protein with important roles in activating T cells especially CD4^+^ T cells to release TNF-α [40,41]. These results collectively help explain the failure of Dasatinib to synergize with ICB in treating CRPC [16]. Therefore, while Dasatinib may block MDSC expansion in certain scenarios [42,43], its adverse effect on T cells [16,44] makes its application in combination immunotherapy unpromising. 

Taken together, our studies highlight the advantage of using a targeted proteomics approach such as RPPA to dissect the impact of inhibitors on primary tumor cells and immune cells. By finding distinct protein expression patterns and differential sensitivity of protein phosphorylation to Dactolisib and Dasatinib, our results offer some in-depth explanations on the molecular basis of the success of Dactolisib/ICB combination and the failure of Dasatinib/ICB combination in the preclinical CRPC model, and strengthen the idea of combining PI3K inhibitors and immunotherapy in treating refractory malignancies. RPPA provides a robust and quick snapshot of some of the most important proteins in cells. Nevertheless, an obvious limitation is that the number of antibodies is still low compared with the entire proteome. Future studies employing mass spectrometry-based proteomics and phosphoproteomics [45] will reveal the effect of targeted therapeutics on Gr-MDSCs in more comprehensive and precise manners. A caveat of the study is that the 2 h treatment of various cells in vitro for RPPA analysis may not represent what these cells experience in vivo when a patient is treated with Dactolisib or Dasatinib. Therefore, conclusions drawn from these in vitro cell models with short drug treatments should be further validated with specimens collected from possible clinical trials that use Dactolisib or Dasatinib to treat patients with PCa.

## 4. Materials and Methods

### 4.1. Mouse Model

*PB-Cre^+^ Pten^L/L^ p53^L/L^ Smad4^L/L^ mTmG^L/+^ LSL-Luc^L/+^* (CPPSML) mice and the method to induce CRPC in male mice were described previously [16]. Briefly, 8–12 weeks old males with correct genotype verified by PCR-based genotyping were imaged by magnetic resonance imaging instrument Bruker ICON (Bruker, Billerica, MA, USA) to quantify prostate tumor volume. Once the prostate tumor reached 150 mm^3^, the mice were surgically castrated and switched to an enzalutamide-admixed diet for 4 weeks. Enzalutamide (MedKoo Biosciences, 201821, Morrisville, NC, USA) was admixed with Purina 5053 Chow at 50 mg drug/kg diet (Research Diets, Inc., New Brunswick, NJ, USA). After 4 weeks, the mice were euthanized for isolating three types of cells. Mice were maintained in pathogen-free conditions. All manipulations were approved by the Institutional Animal Care and Use Committee (IACUC) of University of Texas MD Anderson Cancer Center (Protocol ID 1169, approved on Jan 26, 2015) and University of Notre Dame (Protocol ID 17-04-3809, approved on May 11, 2017). 

### 4.2. Isolation of PCa Cells, Gr-MDSCs and T Cells

CPPSML male mice with CRPC induced were euthanized to harvest prostate tumors and spleens. Prostate tumors were digested to single cells with Mouse Tumor Dissociation Kit (Miltenyi Biotec, 130-096-730, Auburn, CA, USA). Primary PCa cells were isolated by FACS of GFP^+^ CD45^−^ viable cells and subsequently cultured for 2–3 passages in DMEM supplemented with 10% fetal bovine serum (HyClone, GE Healthcare Life Sciences SH30396.03, Marlborough, MA, USA) and 1X Penicillin-Streptomycin (Caisson Labs, PSL01, Smithfield, UT, USA) as adherent primary cells before inhibitor treatment. Intratumoral Gr-MDSCs were isolated by first enriching for lymphocytes using Lympholyte-M Cell Separation Media (Cederlane, CL5031, Burlington, ON, Canada) followed by MACS-based isolation using a Mouse MDSC Isolation Kit (Miltenyi Biotec, 130-094-538, Auburn, CA, USA). From the same mice, total T cells were isolated from the spleens using Mouse Pan T Cell Isolation Kit II (Miltenyi Biotec, 130-095-130, Auburn, CA, USA). Gr-MDSCs and T cells were plated in RPMI-1640 supplemented with 10% fetal bovine serum (HyClone, GE Healthcare Life Sciences SH30396.03, Marlborough, MA, USA) and 1X Penicillin-Streptomycin (Caisson Labs, PSL01, Smithfield, UT, USA) and treated with inhibitors immediately. 

### 4.3. Inhibitor Treatment

Gr-MDSCs, T cells and PCa cells isolated and plated in respective medium as described above were added with DMSO, Dactolisib and Dasatinib at different concentrations for 2 h in a standard tissue culture incubator with 37 °C and 5% CO_2_. Next, the cells were washed with inhibitor-free and serum-free medium once and harvested as pellet and stored at −80 °C before submitting to the RPPA analysis.

### 4.4. Reverse Phase Protein Array (RPPA) 

RPPA was conducted by the RPPA Core Facility at MD Anderson Cancer Center following the standard protocols at the facility, the details of which can be found on the core facility website (www.mdanderson.org/research/research-resources/core-facilities/functional-proteomics-rppa-core.html). The dataset returned from the facility included raw data in log2 value and normalized data in linear value. 

### 4.5. Western Blot

For western blot analysis, cell lysates prepared by the RPPA core were used following the procedure as previously described [16]. Primary antibodies were mostly purchased from Cell Signaling Technology (CST, Danvers, MA, USA), including S6 (CST 2217), phospho-S6 Ser235/236 (CST 4858), phospho-S6 Ser240/244 (CST 5364), 4E-BP1 (CST 9644), phospho-4E-BP1 Thr 37/46 (CST 2855), phospho-4E-BP1 Ser65 (CST 9451), Src (CST 2109), phospho-Src Tyr416 (CST 6943), phospho-Src Tyr527 (CST 2105), p38 MAPK (CST 9212), phospho-P38 MAPK Thr180/182 (CST 4511), Lck (CST 2787), phospho-Lck Tyr394 (Thermo Fisher, PA5-37628, Waltham, MA, USA), phospho-Lck Tyr505 (CST 2751), CD3ε (CST 4443), β-actin (Santa Cruz sc-47778). Secondary antibodies included HRP-linked anti-mouse IgG (CST 7076), HRP-linked anti-rat IgG (CST 7077) and HRP-linked anti-rabbit IgG (CST 7074). Clarity Max western ECL substrate (Bio-Rad, Hercules, CA, USA) was used for chemiluminescence detection.

### 4.6. Microarray and Analysis

RNA was purified from inhibitor treated primary Gr-MDSCs (6 h) using RNeasy Mini Kit (Qiagen, Germantown, MD, USA). Microarray (Affymetrix Mouse Genome 430 2.0 Array) was conducted by the Sequencing and Microarray Facility at MD Anderson Cancer Center following the facility’s standard procedure. The data were analyzed in RStudio, including oligo 1.50.0 package [46] and arrayQualityMetrics 3.42.0 package [47] used for quality control and calibration, ArrayExpress 1.46.0 package [48] and mouse4302.db 3.2.3 package used for probe annotation, and limma 3.42.2 package [49] was used for differential expression analysis. *p* value of 0.05 or 0.01 by unpaired *t*-test was used as threshold of significantly upregulated or downregulated genes by Dactolisib or Dasatinib treatment, respectively. For pathway analysis, the gene lists were imported to RStudio with clusterProfiler 3.14.3 package [50] used for GO term and KEGG pathway analysis and ReactomePA 1.30.0 package [51] used for Reactome analysis. The microarray raw and normalized data were deposited to NCBI GEO database with accession number GSE146083.

### 4.7. Statistical Analysis

Heatmaps were constructed with median-centered log2 values in Figure 1 and normalized log2 values in Figure 2 using package pheatmap 1.0.12 in RStudio. Graphs in Figure 3 were generated using normalized linear values. Significance test in Figure 1C and Figure 5B was by unpaired *t*-test in RStudio.

## Supplementary Materials

Supplementary materials can be found at https://www.mdpi.com/1422-0067/21/7/2337/s1. Supplementary materials include Figure S1. Microarray analysis of Gr-MDSCs. (A) Adjusted-P value distributions for differentially expressed probes between Dactolisib and DMSO control, or between Dasatinib and DMSO control based on unpaired *t*-test, *p* values adjusted by the Benjamini and Hochberg method. (B) KEGG pathway enrichment analysis of downregulated genes by Dactolisib treatment. (C) Reactome enrichment analysis of downregulated genes by Dactolisib treatment; Table S1. RPPA data normalized in linear values, generated by the RPPA Core Facility; Table S2. Differential RPPA protein levels between MDSC and T cells (unpaired student *t*-test); Table S3. Differential RPPA protein levels between MDSC and PCa cells (unpaired student *t*-test); Table S4. Differential RPPA protein levels between PCa cells and T cells (unpaired student *t*-test); Table S5. Microarray gene expression fold changes and *p* values of Dactolisib vs. control treated MDSCs; Table S6. Microarray gene expression fold changes and *p* values of Dasatinib vs. control treated MDSCs; Table S7. GO term over-representation enrichment result of downregulated gene in Dactolisib treated MDSCs based on microarray data; Table S8. KEGG pathway enrichment result of downregulated gene in Dactolisib treated MDSCs based on microarray data; Table S9. Reactome reactions enrichment result of downregulated gene in Dactolisib treated MDSCs based on microarray data.

## Abbreviations

4E-BP1	eukaryotic translation initiation factor 4E binding protein 1
ARID1A	AT rich interactive domain 1A
ATP	adenosine triphosphate
CD3ε	T-cell surface glycoprotein CD3 epsilon chain
CPPSML	*PB-Cre^+^ Pten^L/L^ p53^L/L^ Smad4^L/L^ mTmG^L/+^ LSL-Luc^L/+^*
CRPC	castration-resistant prostate cancer
Csk	C-terminal Src kinase
CTL	cytotoxic T lymphocyte
CTLA4	cytotoxic-T-lymphocyte-associated protein 4
eIF4G	eukaryotic translation initiation factor 4G
FACS	fluorescence-activated cell sorting
Fyn	tyrosine-protein kinase Fyn
GO	Gene Ontology
Gr-MDSC	granulocytic myeloid-derived suppressor cell
ICB	immune checkpoint blockade
KEGG	Kyoto Encyclopedia of Genes and Genomes
Lck	lymphocyte protein tyrosine kinase
MACS	magnetic-activated cell sorting
MAPK	mitogen-activated protein kinase
mTOR	mechanistic target of rapamycin kinase
mTORC1	mTOR Complex I
NDRG1	N-myc downstream regulated gene 1
PCa	prostate cancer
PD1	programmed cell death 1
PD-L1	programmed cell death 1 ligand 1
PI3K	phosphoinositide-3-kinase
PTM	post-translational modification
RPPA	Reverse Phase Protein Array
S6K1	ribosomal protein S6 kinase
Src	Rous sarcoma oncogene
TAZ	tafazzin
TKi	tyrosine kinase inhibitor
TNF-α	tumor necrosis factor alpha
YAP	yes-associated protein
