# Supplementary material for: Differential Targeting of Gr-MDSCs, T Cells and Prostate Cancer Cells by Dactolisib and Dasatinib
Source: Int J Mol Sci. 2020 Mar 27;21(7):2337. doi: 10.3390/ijms21072337 (PMC7178187; doi:10.3390/ijms21072337)
Supplement: Supplementary file 1 [file ijms-21-02337-s001.zip › ijms-752837-Supplementary Materials/ijms-752837-Supplementary Materials.docx]

Supplementary Materials

Supplementary Figure S1


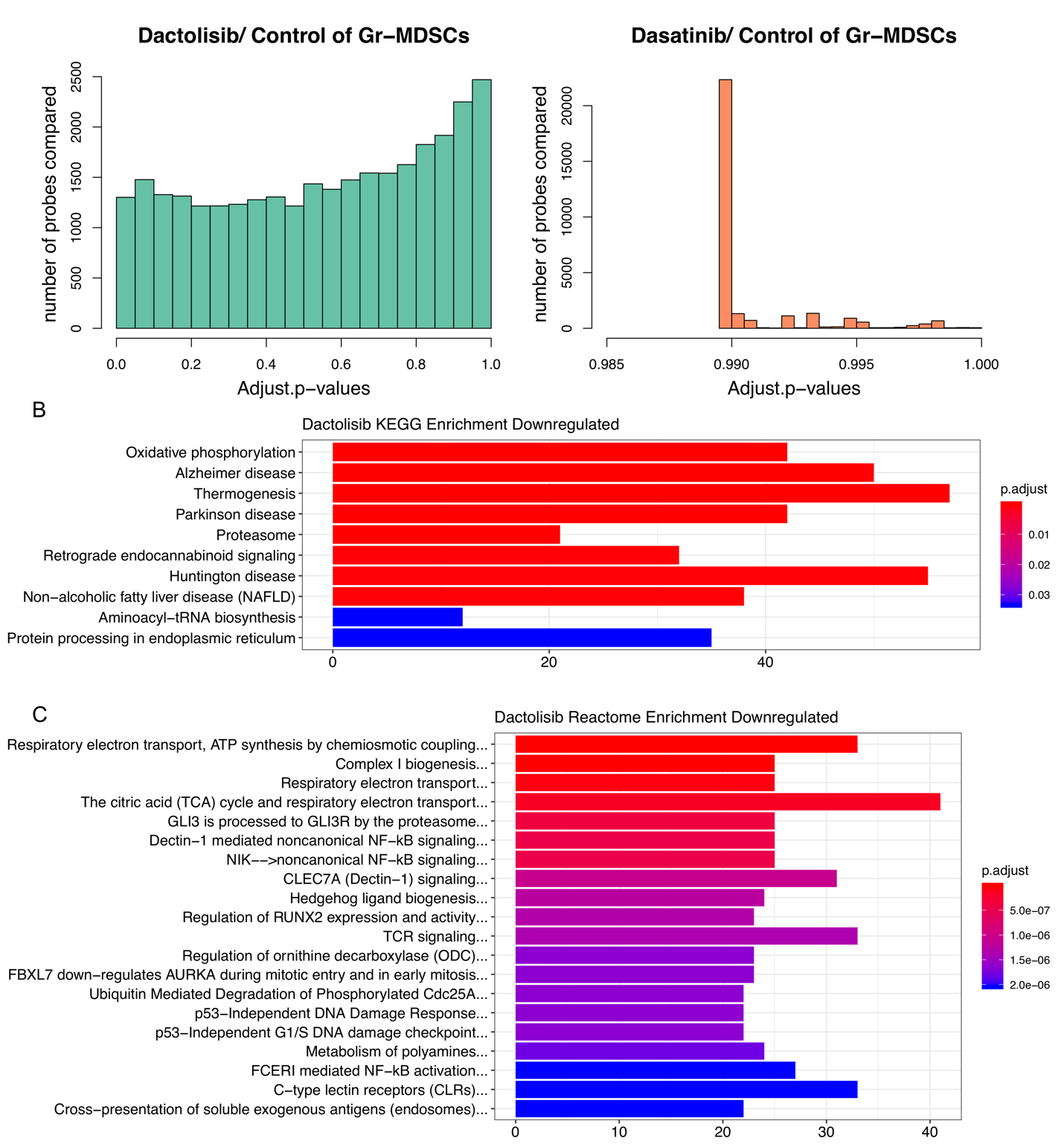


**Figure S1.** Microarray analysis of Gr-MDSCs. **(A)** Adjusted-P value distributions for differentially expressed probes between Dactolisib and DMSO control, or between Dasatinib and DMSO control based on unpaired t-test, P values adjusted by Benjamini and Hochberg method. **(B)**  KEGG pathway enrichment analysis of downregulated genes by Dactolisib treatment. **(C)** Reactome enrichment analysis of downregulated genes by Dactolisib treatment.

**List of Supplementary Tables (Excel spreadsheets):**

**Supplementary Table S1.** RPPA data normalized in linear values, generated by the RPPA Core Facility

**Supplementary Table S2.** Differential RPPA protein levels between MDSC and T cells (unpaired student t test)

**Supplementary Table S3.** Differential RPPA protein levels between MDSC and PCa cells (unpaired student t test)

**Supplementary Table S4.** Differential RPPA protein levels between PCa cells and T cells (unpaired student t test)

**Supplementary Table S5**. Microarray gene expression fold changes and P values of Dactolisib vs. control treated MDSCs

**Supplementary Table S6**. Microarray gene expression fold changes and P values of Dasatinib vs. control treated MDSCs

**Supplementary Table S7.** GO term over-representation enrichment result of downregulated gene in Dactolisib treated MDSCs based on microarray data

**Supplementary Table S8.** KEGG pathway enrichment result of downregulated gene in Dactolisib treated MDSCs based on microarray data

**Supplementary Table S9.** Reactome reactions enrichment result of downregulated gene in Dactolisib treated MDSCs based on microarray data
